# Sequence and expression variations suggest an adaptive role for the *DA1*-like gene family in the evolution of soybeans

**DOI:** 10.1186/s12870-015-0519-0

**Published:** 2015-05-15

**Authors:** Man Zhao, Yongzhe Gu, Lingli He, Qingshan Chen, Chaoying He

**Affiliations:** State Key Laboratory of Systematic and Evolutionary Botany, Institute of Botany, Chinese Academy of Sciences, Nanxincun 20, Xiangshan, 100093 Beijing, China; University of Chinese Academy of Sciences, Yuquan Road 19, 100049 Beijing, China; College of Biological and Environmental Engineering, Zhejiang University of Technology, 310014 Hangzhou, China; College of Agriculture, Northeast Agricultural University, Harbin, Heilongjiang 150030 China

**Keywords:** Evolution, Gene expression, Ortholog, Paralog, Soybean, Transgenic analysis

## Abstract

**Background:**

The *DA1* gene family is plant-specific and *Arabidopsis DA1* regulates seed and organ size, but the functions in soybeans are unknown. The cultivated soybean (*Glycine max*) is believed to be domesticated from the annual wild soybeans (*Glycine soja*). To evaluate whether *DA1-*like genes were involved in the evolution of soybeans, we compared variation at both sequence and expression levels of *DA1*-like genes from *G. max* (*GmaDA1*) and *G. soja* (*GsoDA1*).

**Results:**

Sequence identities were extremely high between the orthologous pairs between soybeans, while the paralogous copies in a soybean species showed a relatively high divergence. Moreover, the expression variation of *DA1*-like paralogous genes in soybean was much greater than the orthologous gene pairs between the wild and cultivated soybeans during development and challenging abiotic stresses such as salinity. We further found that overexpressing *GsoDA1* genes did not affect seed size. Nevertheless, overexpressing them reduced transgenic *Arabidopsis* seed germination sensitivity to salt stress. Moreover, most of these genes could improve salt tolerance of the transgenic *Arabidopsis* plants, corroborated by a detection of expression variation of several key genes in the salt-tolerance pathways.

**Conclusions:**

Our work suggested that expression diversification of *DA1*-like genes is functionally associated with adaptive radiation of soybeans, reinforcing that the plant-specific *DA1* gene family might have contributed to the successful adaption to complex environments and radiation of the plants.

**Electronic supplementary material:**

The online version of this article (doi:10.1186/s12870-015-0519-0) contains supplementary material, which is available to authorized users.

## Background

The cultivated soybean (*Glycine max*) is a staple crop offering multiple proteins and oils for humans. It was suggested that the cultivated soybean was domesticated from its annual wild relative *Glycine soja*. Wild soybeans have a wide geographical distribution, and a wide range of genetic variations have been accumulated with adaption to geographic, abiotic and biotic environmental conditions during evolution [1, 2]. Although the domestication process has endowed cultivated soybeans with many advantages in morphological and physiological traits, studies have revealed that wild soybeans had more genetic diversity, especially for stress resistance, than the soybean cultivars [3]. Therefore, the wild soybean germplasm should be a potential genetic reservoir for the improvement of cultivated soybeans. It is an effective way to facilitate agricultural development through exploring and importing excellent genes from wild species [4]. Several agricultural traits of wild soybeans have already been introduced into cultivated soybeans [5, 6].

Salt stress has posed a great threat to agricultural development around the world. Soil salinity can inhibit plant growth and decrease crop yields [7]. More than 800 million hectares of lands have been affected by salinity per year in the world [8]. In *Arabidopsis thaliana*, the ionic signaling pathway is transduced via the salt-overly-sensitive (SOS) pathway where a calcium-responsive SOS3-SOS2 protein kinase complex controls the expression and activity of ion transporters [9, 10]. The complex of serine/threonine protein kinase SOS2 and the myristoylated calcium-binding protein SOS3 can be activated by a salt-stress-elicited calcium signal, and then the activated protein kinase complex phosphorylates and activates various ion transporters, such as the plasma membrane Na^+^/H^+^ antiporter SOS1 [10], which improves salt-tolerance. In response to osmotic stress caused by salt, plants can accumulate metabolites that act as compatible solutes to lower the cellular osmotic potential without affecting normal metabolic reactions [11]. Delta 1-pyrroline-5-carboxylate synthetase gene (*P5CS1*) encodes the rate-limiting enzyme involved in proline synthesis [12, 13]; this enzyme can increase the rate of proline synthesis, which results in proline accumulation and increased osmotic pressure, thus increasing salt tolerance in plants [14]. Some other genes, such as the alcohol dehydrogenase gene (*ADH*) and *FIERY1* (*FRY1*) gene, that were involved in salt tolerance were related to the phytohormone abscisic acid (ABA) [15, 16]. Salt tolerance mechanisms in soybeans are unknown, but they have been proposed to consist of the maintenance of ion homeostasis, adjustment in response to osmotic stress, restoration of oxidative balance and other metabolic and structural adaptations [17].

*Arabidopsis DA1* and *DA1*-related 1 (*DAR1*) genes, as representatives of the *DA1* gene family, have a function in regulating organ and seed size [18, 19]. The *DA1* expression is induced by ABA [18], implying that the *DA1*-like genes may be involved in the response to abiotic stresses. This assumption has been further corroborated by observations of another *DAR* gene; i.e., the *chilling sensitive 3* (*CHS3*) gene, which plays a role in biotic and abiotic resistance responses [20, 21]. Therefore, *DA1*-like genes have multiple functions in plants. A genome-wide evolutionary study suggested that the *DA1* gene family encodes a group of proteins belonging to the LIM (from lin-11, isl-1 and mec-3) domain superfamily, and that this gene family is plant-specific and may play a role in the successful radiation of plants in diverse environments during evolution [22].

Since wild soybeans are distributed in a variety of adverse natural environments, they may act as a source for improving phenological, agronomic traits, and the resistances and tolerances to biotic and abiotic stresses of cultivated soybeans [2, 23]. Comparative study of wild and cultivated soybeans at gene, genomic and transcriptomic levels might provide multiple genetic resources to improve the yield and quality of cultivated soybeans. Such studies will also provide valuable insights into the molecular basis of soybean domestication, in turn further directing genetic improvement of modern soybean cultivars. Whether the *DA1*-like genes were involved in the evolution / domestication of soybeans is unknown. In the present study, we evaluated this potentiality via comparison of sequences and expressions of the *DA1* gene family between *G. max* (*GmaDA1*) and *G. soja* (*GsoDA1*). Little sequence divergence was observed between the Gma-GsoDA1 orthologous protein pairs while a relative high divergence was found among paralogous proteins in a species. The expression of their genes variously responded to diverse abiotic stresses. Moreover, overexpressing some *GsoDA1* genes could improve salt-tolerance of the transgenic *Arabidopsis* plants. Therefore, our results suggested that expression variation of the *DA1*-like genes may be largely attributed to the adaptive evolution of soybeans.

## Results

### Sequencing analyses of the *GmaDA1* and *GsoDA1* genes

**Fig. 1:**
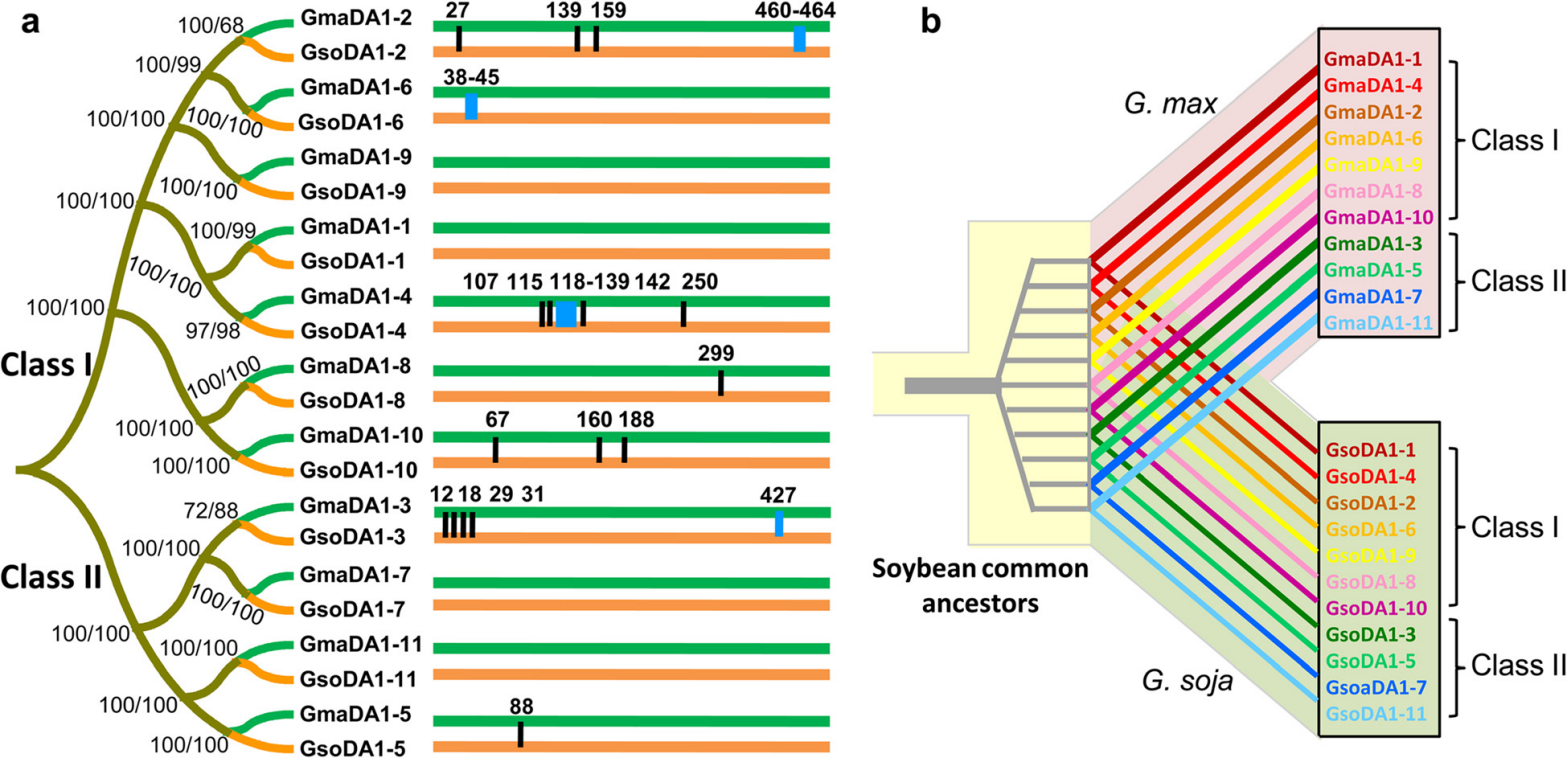
The phylogenetic relationship and sequence divergence of DA1-like proteins from *G. soja* (GsoDA1) and *G. max* (GmaDA1). **a** The ML (maximum likelihood) and NJ (neighbor-joining) trees were constructed using protein sequences. The green lines represent GmaDA1 proteins, and the orange lines represent GsoDA1 proteins. The position of amino acid substitution sites (vertical black lines) and of indels (vertical blue lines) is shown (details in Additional file 1: Table S2). **b** Schematic representation of the evolutionary process of *DA1*-like genes during the speciation of soybeans

The soybean genome encodes 11 *DA1*-like genes [22]. We first cloned their cDNAs in the cultivated soybean Suinong 14 and the wild accession ZYD00006. To show the orthology between the cultivated and the wild soybeans, an unrooted phylogenetic tree was constructed using the amino acid sequences. In line with our previous work [22], the *DA1*-like genes in soybeans diverged into Class I and Class II (Fig. 1a). For each member, the two sequences from the cultivated and wild soybeans gathered together to form a pair (Fig. 1a), suggesting their orthology, and their inheritance from common ancestors (Fig. 1b). The sequence identities of the 11 orthologous pairs ranged from 95.8 to 100 % (Additional file 1: Table S1), indicating a high conservation of these orthologous pairs. Four orthologous pairs were identical in Class I (DA1-1 and DA1-9) and Class II (DA1-7 and DA1-11), and the rest contained a few amino acid substitutions and indels (Fig. 1a; Additional file 1: Table S2). Nonetheless, sequence identities of DA1-like paralogs in a soybean showed a large difference ranging from 44.1 to 97.7 % (Additional file 1: Table S1). These results suggested that the 11 DA1-like paralogs in a soybean species originated in soybean common ancestors (Fig. 1b) became more diversified than the orthologous pairs between the cultivated and wild soybeans during evolution.

To estimate the potential effects of amino acid substitutions on protein functions, we performed SNAP and PROVEAN analyses (see Methods). The results of SNAP showed that all substitutions were neutral with an expected accuracy of more than 60 %, suggesting that these substitutions would be unlikely to change the protein functions (Additional file 1: Table S2). Furthermore, the PROVEAN analysis also predicted that almost all of the substitutions might not influence protein functions except for position 299 in DA1-8. The predicted score of substitution in DA1-8 was −5.7, which was less than the cutoff value (−2.5), implying that the substitution may be deleterious to the protein functions (Additional file 1: Table S2). The inconsistent prediction of DA1-8 in these two analyses may be caused by different referential databases of the two methods. Besides the amino acid substitutions, the indels could also affect the protein functions. Therefore, we used PROVEAN analysis to predict the functions of these indels, and showed that all the indels were neutral. Meanwhile, prediction of subcellular locations showed that there was no divergence in the distributions of these Gma-GsoDA1 orthologous pairs. Nonetheless, subcellular locations of different DA1-like members (paralogs) in a soybean species were diverged (Additional file 1: Table S2). These predictions need to be further clarified experimentally, but they may reflect the putative functional variation resulting from the coding sequence variation in these *DA1*-like genes. Besides this, the divergence of *DA1*-like genes may occur in their expression levels.

### The *DA1*-like expression in various organs of soybeans

**Fig. 2:**
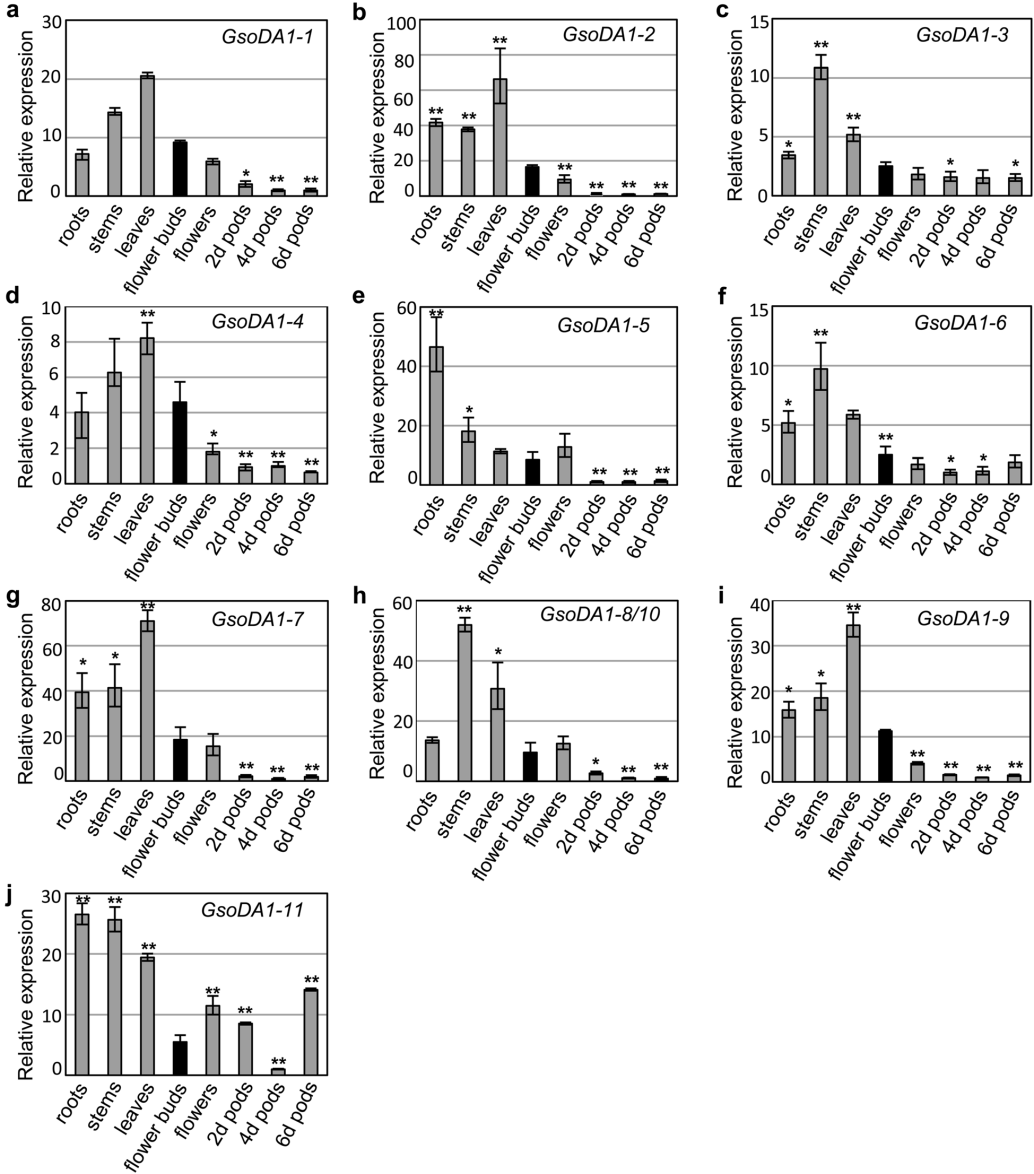
Expression of the *GsoDA1* genes during soybean development. (**a-j**) The expression of *GsoDA1-1* (**a**), *GsoDA1-2* (**b**), *GsoDA1-3* (**c**), *GsoDA1-4* (**d**), *GsoDA1-5* (**e**), *GsoDA1-6* (**f**), *GsoDA1-7* (**g**), *GsoDA1-8/10* (**h**), *GsoDA1-9* (**i**) and *GsoDA1-11* (**j**). The total RNAs were isolated from roots, stems, leaves of 14-day-old seedlings, unfertilized flower buds, flowers, and 2-, 4- and 6-day-old fruits after fertilization. The *ACTIN* gene was used as an internal control. The experiments were repeated using three independent biological samples. Error bar: standard deviation. The significance was tested in comparison with the expression of each gene in flower buds (black column). The * means significance at a *P* < 0.05 level, and the ** represents the significance at a *P* < 0.01 level

qRT-PCR was used to detect the relative expression levels of *DA1*-like genes in various tissues of soybeans. Owing to high sequence identities, the degenerate primers of *DA1-8* and *DA1-10* were designed and named *DA1-8/10*. The tissue-specific expression of *GmaDA1* genes in the cultivated soybean Suinong 14 was previously investigated [22]. Here we investigated the expression of *GsoDA1* genes in various organs in wild soybean ZYD00006 (Fig. 2). *GsoDA1* genes presented a higher expression level in roots, stems and leaves than the reproductive organs such as the flowers and the developing fruits. Moreover, the expression levels of these genes overall declined during fruit development with few fluctuations. The overall extensive expression of the *DA1*-like genes in various organs implied that they may have universal roles in soybean development. We further compared the expression profile of *GmaDA1* [22] and *GsoDA1* (Fig. 2) orthologous gene pairs and we did not find a significant divergence (*P* > 0.09) between these 11 orthologous gene pairs, while *GmaDA1* (*P* = 0.009) or *GsoDA1* (*P* = 0.04) paralogs in a species were different in all the detected organs, indicating an organ-biased role for each soybean *DA1*-like gene.

### Soybean *DA1-*like expression in response to various abiotic stresses

**Fig. 3:**
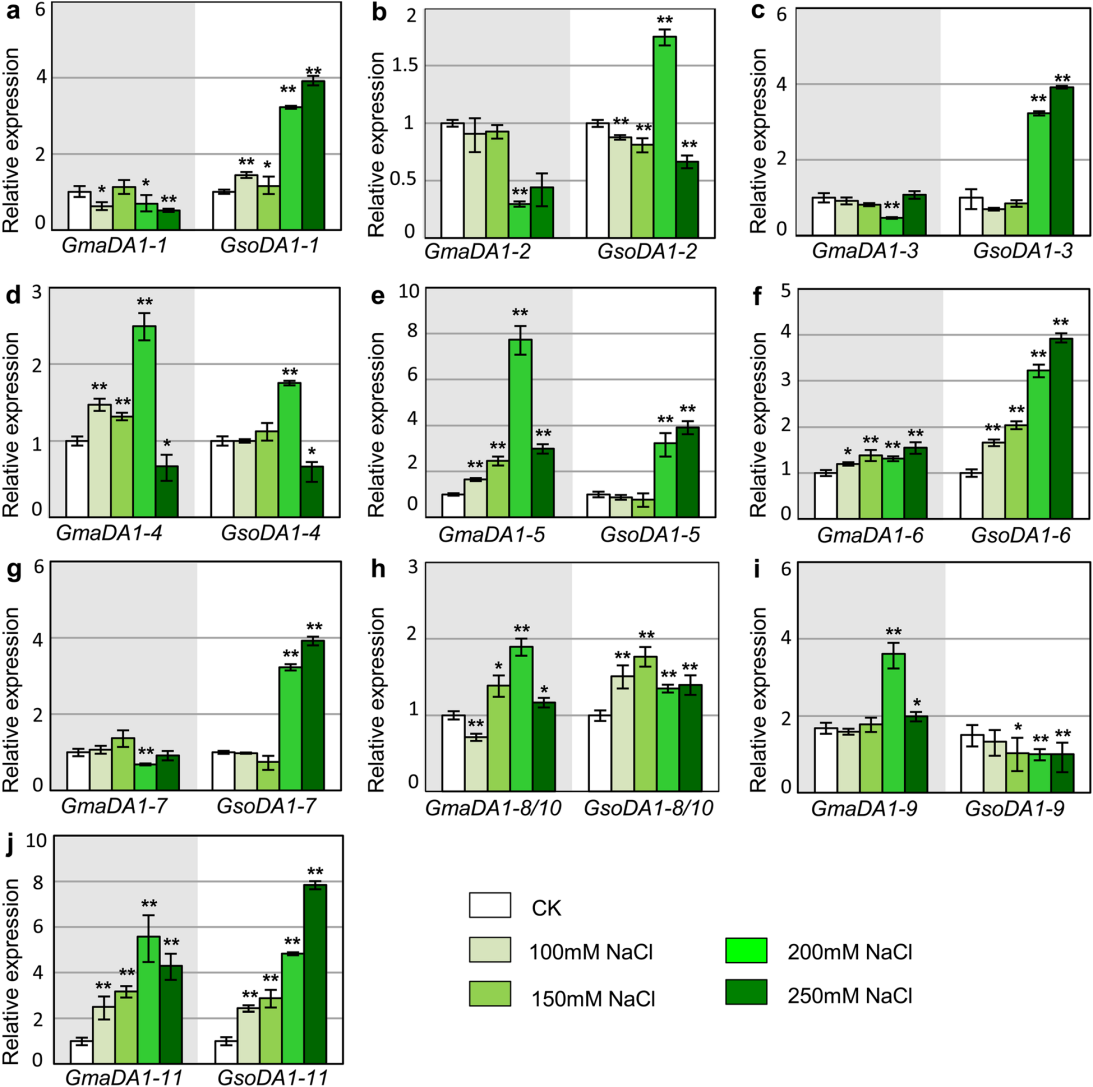
Expression of the *GmaDA1* and *GsoDA1* genes in response to salt stress. (**a-j**) The expression of *Gma-GsoDA1* orthologous gene pairs: *DA1-1* (**a**), *DA1-2* (**b**), *DA1-3* (**c**), *DA1-4* (**d**), *DA1-5* (**e**), *DA1-6* (**f**), *DA1-7* (**g**), *DA1-8/10* (**h**), *DA1-9* (**i**) and *DA1-11* (**j**). Total RNAs from roots after 4 h treatment for different NaCl stresses were subjected to qRT-PCR analyses. Expression of each gene in the non-treated conditions (white column) was used as a control (CK) and was set as 1. The expression of *GmaDA1* challenging 200 mM NaCl has been reported [22]. The *ACTIN* gene was used as an internal control. The experiments were performed using three independent biological samples. Error bar: standard deviation. The * means significance at a *P* < 0.05 level, and the ** represent the significance at a *P* < 0.01 level. For easy comparison of *Gma-GsoDA1* orthologous gene pairs, the *GmaDA1* expression was displayed in a gray ground

We next investigated the potential evolutionary patterns of expression variation of soybean *DA1-*like genes in adaptation to natural environments, fulfilled by applying various external stimuli to the soybean seedlings. Divergence of *GmaDA1* and *GsoDA1* genes in response to salt stresses was first analyzed using qRT-PCR (Fig. 3). Overall, the expression of *Gma-GsoDA1* orthologous pairs was relatively conserved except for *DA1-8/10* (*P* = 0.02) when challenged with different strengths of salt stresses. Considering the details, these genes actually showed different patterns in tendency and magnitude in response to salt stresses. Some *Gma-GsoDA1* orthologous gene pairs shared a similar expression variation pattern, such as *DA1-4*, *DA1-5*, *DA1-6*, *DA1-8/10* and *DA1-11* (Fig. 3d–f, h, j). The expression level of *DA1-4* was elevated with a peak at 200 mM (*P* < 0.002), but was down-regulated at 250 mM in the wild and cultivated soybeans (*P* < 0.03; Fig. 3d). *DA1-6* and *DA1-11* were consistently induced in wild and cultivated soybeans (*P* < 0.01; Fig. 3f, j). *DA1-5* and *DA1-8/10* were also significantly induced in soybeans to different extents (*P* < 0.002; Fig. 3e, h). We also observed different expression variations between *Gma-GsoDA1* gene pairs under different strengths of salt stress. The most striking pattern was that the expression trends of the orthologous gene pairs were opposite and showed that the expressions of *DA1-1*, *DA1-3* and *DA1-7* were significantly suppressed in cultivated soybeans, but were intensely induced in wild soybeans (Fig. 3a, c, g), and the *DA1-9* orthologous pair had an opposite tendency (Fig. 3i), suggesting a divergence in *Gma-GsoDA1* orthologous gene pairs in response to salt stresses. Nevertheless, the variation extents were different in different genes. For example, *GmaDA1-3* and *GmaDA1-7* were repressed only at 200 mM NaCl (*P* < 0.006), but *GmaDA1-1* was declined (*P* < 0.03) except for the 150 mM NaCl stress condition (*P* = 0.25)*. GsoDA1-3* and *GsoDA1-7* were only increased in 200 mM and 250 mM (*P* < 0.0001), but *GsoDA1-1* was elevated under all the NaCl concentrations (*P* < 0.03). The *DA1-2* gene showed complex and different expression variations in the two soybeans (Fig. 3b). With the salt concentration increasing, *GmaDA1-2* was basically unchanged (*P* > 0.12) except for a decline at 200 mM (*P* = 0.003), but the *GsoDA1-2* was basically repressed except for a peak at 200 mM (*P* < 0.009). In a dramatic contrast, the *GsoDA1* or *GmaDA1* paralogs in a soybean showed extremely diverse variations in both tendency and magnitude of gene expression (*P* < 0.02). These observations suggested that the *DA1* genes might have different roles in response to salt, and they may have diverged in wild and cultivated soybeans.

Besides salinity, these genes also differentially responded to drought, acidic and alkali-stresses and ABA treatment (Additional file 1: Dataset S1; Additional file 1: Figures S1–S3). Thus, the expression variation of soybean *DA1*-like genes in response to various stresses suggested that they might also exert roles in stress-tolerance in soybeans associated with the adaptation evolution of soybeans.

### Overexpressing *GsoDA1* affected seed germination under salinity

**Fig. 4:**
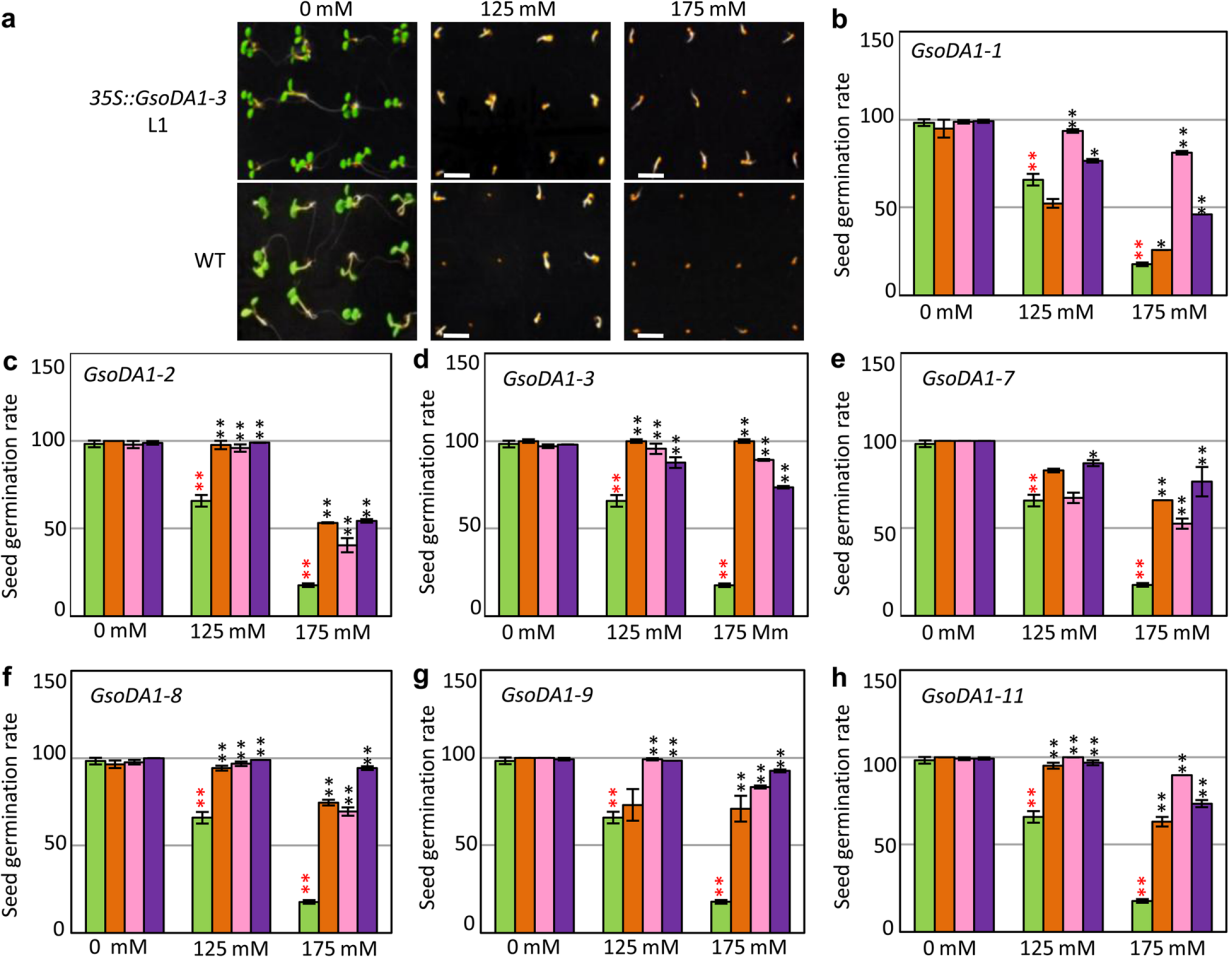
Determination of seed germination rates of transgenic *Arabidopsis* plants (**a**) Seeds of the wild-type (WT) and the L1 transgenic *Arabidopsis* plants (*35S::GsoDA1-3*) were germinated for five days. These seeds were treated by 0 mM, 125 mM and 175 mM NaCl, respectively. Bars = 2.5 mm. **b-h** Germination rates of WT and transgenic *Arabidopsis* seeds under salinity. The green column represents the WT, and the orange, pink and purple columns represent the transgenic lines L1, L2 and L3, respectively. Error bar: standard deviation. The red ** represent the significance at a *P* < 0.01 level in comparison to WT without treatment. The black * means significance at a *P* < 0.05 level, and the black ** represents the significance at a *P* < 0.01 level in comparison with WT at the same condition

To get further clues about the role of *DA1*-like genes in the development and the adaptation of soybeans, we overexpressed some of these genes in *Arabidopsis. GsoDA1-1*, *GsoDA1-2*, *GsoDA1-3*, *GsoDA1-7*, *GsoDA1-8*, *GsoDA1-9* and *GsoDA1-11* were manipulated and three independent T_3_ transgenic *Arabidopsis* plants for each *GsoDA1* gene were analyzed (Additional file 1: Figure S4). The phenotypic variations of transgenic lines of each *GsoDA1* gene, such as seedling morphology, seed size and germination rate, were not deviated from those of the wild-type *Arabidopsis* under normal conditions (Additional file 1: Figure S5; Fig. 4a). However, different seed germination capabilities of transgenic plants from the wild-type seeds were observed in salinity conditions. Vernalization was performed to synchronize seed germination. No significant difference in seed germination was observed between transgenic plants and the wild-type *Arabidopsis* growing under normal conditions (Fig. 4). The germination rate of the wild-type seeds was significantly reduced under salinity conditions (green column, red stars in Fig. 4b–h); however, the transgenic *Arabidopsis* seeds on salt-containing medium had statistically higher germination rates than the wild-type *Arabidopsis* seeds (black stars in Fig. 4b–h). Thus, overexpression of *GsoDA1* genes could reduce the sensibility of seed germination to salt in transgenic *Arabidopsis*.

### *GsoDA1* transgenic *Arabidopsis* plants showed an enhanced salt tolerance

**Fig. 5:**
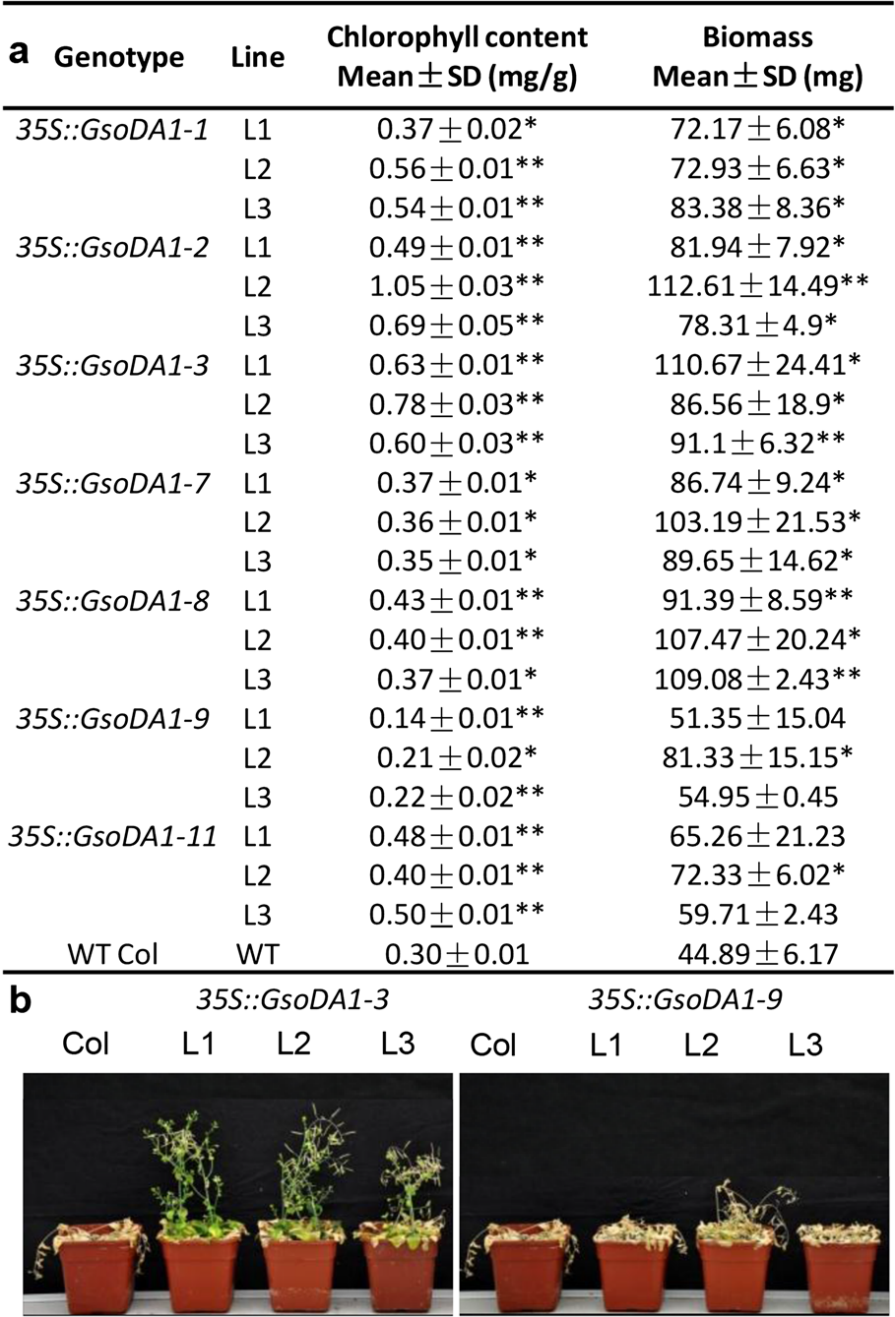
Improved salt tolerance of *GsoDA1*-transgenic plants **a**) Determination of chlorophyll content after the 15th-day treatment andmeasurement of the aboveground biomass after the 30th-day treatment. SD means standard deviation. The star means significant difference between the transgenic plants and wild type *Arabidopsis.* The * and ** represent the significance at a *P* < 0.05 level and at a *P* < 0.01 level, respectively. **b** Comparison of *GsoDA1*-transgenic and wild-type plants under salt stress. The 4-week-old seedlings of transgenic and wild-type plants were treated with 250 mM NaCl. Plants were photographed after treatment for 20 days. Two representatives are shown. Left: *35S::GsoDA1-3*, right: *35S::GsoDA1-9*

Salt tolerance of *GsoDA1* transgenic plants was further evaluated. Four-week old seedlings were watered with 250 mM saline solution (one time on five days). The wild-type control plants and *35S::GsoDA1-9* transgenic lines came to exhibit much more bleaching and wilt than other transgenics under the same salt treatment after 15 days of treatment. Hence, chlorophyll contents were determined in wild-type controls as well as in transgenic plants at this time. The chlorophyll content of *35S::GsoDA1-9* transgenics was significantly reduced, while a relatively and significantly high content of chlorophyll was observed in other salt-tolerant transgenic plants in comparison to the wild-type control plants (Fig. 5a). In line with this, we observed that the aboveground biomasses of all the transgenic *GsoDA1-1*, *GsoDA1-2*, *GsoDA1-3*, *GsoDA1-7* and *GsoDA1-8* plants were significantly enhanced; nonetheless, no significant difference in biomasses was observed between the wild-type control and most transgenic lines of *35S::GsoDA1-9* and *35S::GsoDA1-11* under salinity conditions (Fig. 5a). Around the 20th-day treatment, the wild-type *Arabidopsis* and the transgenic lines harboring *GsoDA1-9* died, while the other *35S::GsoDA1* transgenic *Arabidopsis* plants, *35S::GsoDA1-3* plants as an example, survived (Fig. 5b). Thus, overexpressing *GsoDA1* genes might reduce salt injury to plants, and improve the salt tolerance of plants.

### The salt-tolerance pathway was affected in *GsoDA1* transgenic *Arabidopsis* plants

**Fig. 6:**
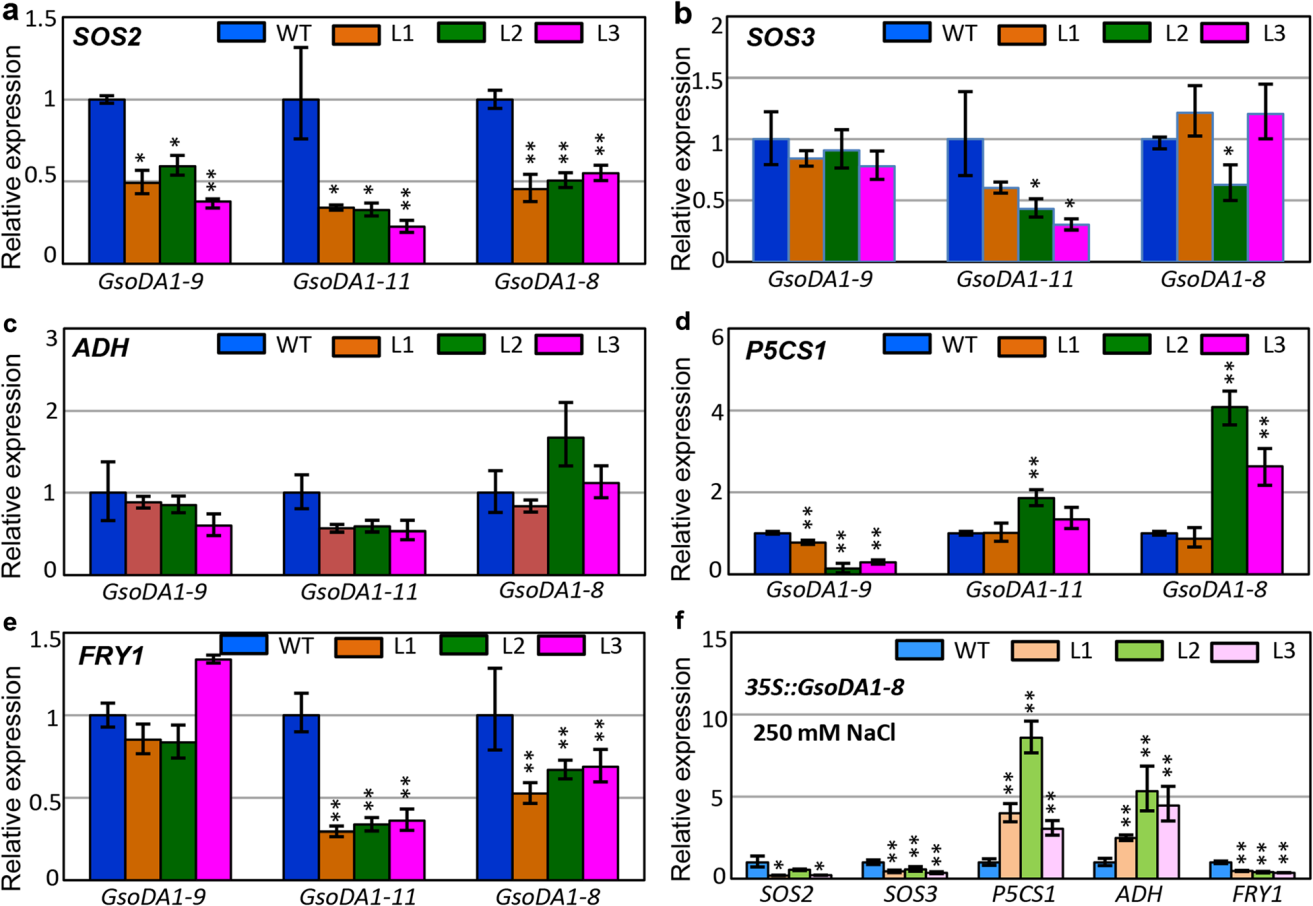
Expression of salt tolerance-related genes in wild-type and transgenic *Arabidopsis.* **a–e** The expression variation of *SOS2* (**a**), *SOS3* (**b**), *ADH* (**c**), *P5CS1* (**d**) and *FRY1* (**e**) in the indicated transgenic *Arabidopsis* lines in comparison with wild-type *Arabidopsis* (WT). **f** The *35::GsoDA1-8* transgenic *Arabidopsis* seedlings were also treated by 250 mM NaCl. Expressions of each gene in wild-type *Arabidopsis* (dark blue and light blue columns) were set as controls (CK). The *ACTIN* gene was used as an internal control. The experiments were performed using three independent biological samples. Error bar: standard deviation. The * means significance at a *P* < 0.05 level, and the ** represent the significance at a *P* < 0.01 level

To further understand the role of *GsoDA1* genes in salt stress signaling pathways, the expression of *SOS2*, *SOS3*, *ADH*, *P5CS1* and *FRY1* genes, which are known in salt tolerance pathways, were investigated in transgenic plants. Transgenic *Arabidopsis* plants of *35S::GsoDA1-8*, *35S::GsoDA1-9* and *35S::GsoDA1-11* were compared with the wild-type *Arabidopsis*. The *SOS2* expression in all these transgenic lines was significantly down-regulated (Fig. 6a). The *SOS3* expression in *35S::GsoDA1-11* transgenic lines was reduced (Fig. 6b). The *ADH* expression in these transgenic plants were not statistically changed (Fig. 6c). The *P5CS1* expression in different lines of *35S::GsoDA1-9* transgenic plants were significantly suppressed (Fig. 6d). The expression of *FRY1* was significantly suppressed in the *35S::GsoDA1-11* and *35S::GsoDA1-8* transgenic *Arabidopsis* plants but not changed in the *35S::GsoDA1-9* transgenic *Arabidopsis* plants (Fig. 6e). We further investigated the effect of *GsoDA1* genes on these salt-tolerance genes in transgenic *Arabidopsis* plants under salinity conditions. For this purpose, *35S::GsoDA1-8* transgenic plants were treated with 250 mM NaCl. The expression of *SOS2*, *SOS3* and *FRY1* genes was further repressed by salt stress, while both *ADH* and *P5CS1* expressions were enhanced (Fig. 6f). Thus, overexpressing different *GsoDA1* genes differentially affected the expression of salt-tolerance related genes in transgenic *Arabidopsis* plants.

## Discussion

The *DA1* gene family is plant-specific functioning in organ size control [18, 19] and has been suggested to play multiple roles in plants during plant development and evolution [22]. To further expand this knowledge, in the present study, we comparatively investigated the sequences and expression patterns of the *DA1* gene family in cultivated and wild soybeans, providing further insights into the roles of this gene family in soybean development and evolution.

### Soybean *DA1* genes have roles in challenging stresses

ABA plays essential roles in many physiological processes, such as embryogenesis, seed dormancy, leaf transpiration and stress tolerance [24, 25]. The expression of *Arabidopsis DA1* was induced by ABA [18] and soybean *GmaDA1* genes also responded to various stresses, including ABA [22], suggesting that *DA1*-like genes might be involved in multiple developmental and physiological processes. In the present study, we found that all these genes from *G. max* and *G. soja* were substantially variably expressed in response to different abiotic stresses and ABA, further suggesting a role for these genes related to stress tolerance.

Salt stress is one of the main abiotic stresses that significantly inhibits plant growth and reduces crop production. The mechanism underlying salt tolerance has been extensively investigated in the model plants *Arabidopsis* and rice [10, 26–28]. However, owing to ancient allotetraploid with a large and highly duplicated genome, the absence of an extensive mutant collection and the inefficient transformation system of soybeans, the information available on salt stress responses and possible tolerance mechanisms in soybean is preliminary [29, 30], and such roles for soybean genes are usually inferred from transgenic *Arabidopsis* [31, 32]. Although the link between gene expression in response to stresses in soybeans and phenotypic variations (in morphology, gene expression and physiology) of transgenic *Arabidopsis* harboring cDNA of each gene could not be clearly established in the present work, our transgenic *Arabidopsis* analyses revealed that overexpressing some *GsoDA1*-like genes could improve the salt-tolerance of transgenic *Arabidopsis* plants, suggesting that the soybean *DA1*-like genes may be involved in the salt tolerance signal pathways. Furthermore, we found that the expressions of *SOS2* [10], *SOS3* [10], *ADH* [15], *P5CS1* [14] and *FRY1* [16] genes, which were involved in the salt-tolerance pathways, were differentially affected in *GsoDA1* transgenic *Arabidopsis* plants in a complex manner. The downregulation of *FRY1* and the elevated expression of *SOS2*, *SOS3*, *ADH* and *P5CS1* can increase salt tolerance in plants [10, 14–16]. Therefore, the alteration of these genes might, in part, account for the alteration of salt-tolerance of transgenic *Arabidopsis* plants. *Arabidopsis DA1*-like genes were found to be involved in the ubquitination processes [18, 19], while how they affected gene expression at a transcription level remained unknown. Our observations further suggested that different *DA1* paralogs in a soybean species might be involved in different stress signal pathways. Nonetheless, the role of soybean *DA1*-like genes in both development and challenging stresses needs further substantiation in native hosts.

### The role of the *DA1* gene family in the evolution of soybeans

Wild and cultivated soybeans were regarded to have common progenitors, and the domestication has endowed the cultivated soybean with many advantages in morphological and physiological traits [3], including retention of seed on the seed head, reduction in lateral branching, reduction in seed dormancy, an increase in seed size, and shifts in flowering time and grain composition [33]. A growing number of major-effect domestication and crop improvement genes have been investigated, including the *indeterminate* gene *1* (*Dt1*) in soybean [34], *fascinated* gene (*fas*) in tomato [35], *shattering* gene *1* (*Sh1*) in sorghum [36] and *FLOWERING LOCUS T* gene (*HaFT1*) in sunflower [37]. These studies revealed a diversity of underlying causative mutations affecting phenotypes important in plant domestication, including coding sequence substitutions, copy number variation, transposon activation leading to novel gene structures and expression patterns, diversification following gene duplication, and polyploidy leading to altered combinatorial capabilities [38]. To exploit the role of *DA1*-like genes in soybean domestication, we comparatively analyzed the sequences and expressions of these genes among cultivated and wild soybeans. Although *Arabidopsis DA1*-like genes are involved in seed size control [18, 19], we did not find any evidence supporting such a role for *DA1*-like genes in soybeans. The differential expressions of *DA1-6*, *DA1-8/10* and *DA1-11* between the cultivated and wild soybeans in the reproductive organs hinted at a potential role in seed development, but the transgenic *Arabidopsis* plants harboring *GsoDA1* genes did not affect seed size. Manipulating *GmaDA1* genes in transgenic *Arabidopsis* might clarify this. Nonetheless, the role of *DA1*-like genes in organ size control might be specific to the *Brassica* species or be a gain-of-function related to the *da1-1* mutation (R358K) in *Arabidopsis* [18], since all isolated soybean *DA1*-like genes encoded putative proteins having a conserved 358 site (R).

Domesticated genes often have large phenotypic effects and are relatively insensitive to genetic background and environmental effects [33]. However, human selections put the progenitors of the cultivated soybeans in conditions that were totally different from natural conditions, thus effects in the physiological response to stresses during soybean domestication could be estimated. The sequences of Gma-GsoDA1 orthologous gene pairs originated from common ancestors were extremely conserved, and their mRNA expression profiles during development were similar overall. However, responses of some orthologous gene pairs to different abiotic stresses were different, indicating potential effects of domestication on soybean genomes. Both sequences and expression among the *DA1-*like paralogs in a soybean species changed dramatically. In particular, the expression variation in response to various stresses was tremendous. These variations might have contributed to soybean adaptation during evolution. This assumption was further supported by our observations that the transgenic *Arabidopsis* harboring *GsoDA1* genes showed enhanced salt-tolerance. The underlying evolutionary mechanisms of these sequence and expression variations need further investigation in population level of both *G. max* and *G. soja*; nonetheless, the *DA1*-like genes seem to be involved in the adaptive evolution of soybeans.

## Conclusions

Soybean *DA1*-like genes were inherited from common ancestors, and comparative studies of the *DA1* gene family in wild and cultivated soybeans have suggested that the sequences of *DA1*-like orthologous pairs are more conserved than the paralogous copies in soybean species, but the expression of these genes varies when challenging different abiotic stresses. Overexpressing *GsoDA1* genes did not alter seed sizes of transgenic *Arabidopsis*, but improved salt tolerance of the transgenic plants, thus elevating expression of some genes may improve stress-tolerance of soybeans. The observed divergence of the *DA1*-like genes in cultivated soybean compared to wild soybean is part of the genetic variation influenced by human breeding, while the variations in paralogous copies in a species, particularly in wild soybean are largely associated with the evolution of soybeans under natural environments. This study further suggested that the plant-specific *DA1* gene family might have contributed to the successful adaption to complex environments and radiation of the plants.

## Methods

### Plant materials and growth conditions

The cultivated soybean ‘Suinong14’ and the wild accession ‘ZYD00006’ were grown in a greenhouse of the Institute of Botany (Beijing, China) under short-day conditions (16 h dark / 8 h light at 23–25 °C). The flower buds, mature flowers, and 2-, 4- and 6-day post-fertilization fruits were harvested at same time point to study gene expression. To evaluate gene expression in response to stresses, soybeans were cultured with 50 % Hoagland solution in a growth chamber under long-day conditions (16 h light / 8 h dark at 23–25 °C). The roots, stems and leaves were collected from 2-week-old seedlings at same time point. The harvested tissues were immediately stored in liquid N_2_ and then stored at −80 °C for total RNA extraction using TRIzol reagent (Invitrogen).

### Abiotic stress treatments

For stress treatment, two-week-old ‘Suinong14’ and ‘ZYD00006’ seedlings growing in a chamber were transferred to modified 50 % Hoagland solution containing NaCl (100 mM, 150 mM, 200 mM or 250 mM), polyethylene glycol (PEG6000 10 %, 15 %, 20 % or 25 %), acids (pH2, pH3, pH4 or pH5), and alkaline (pH7, pH8, pH9 or pH10) for 4 h, respectively. To analyze ABA responsiveness, the seedlings were transferred to the Hoagland solution containing 10 μM ABA for 1 h, 3 h, 6 h or 12 h. Soybean seedlings without any treatment were used as controls. The roots were harvested at the appropriate times for expression studies.

### Quantitative RT-PCR analyses

Two micrograms of total RNA were treated with DNase I (Sigma-aldrich, USA) and used to synthesize the first strand cDNA using a M-MLV cDNA Synthesis Kit (Invitrogen). Quantitative RT-PCR (qRT-PCR) was conducted using SYBR Premix Ex Taq™ (TaKaRa) in an Mx3000P QPCR system (Stratagene). *ACTIN* (Glyma.18G290800) was used to as an internal control. Each experiment was performed using three independent biological samples. PCR was performed in a 25.0 μL reaction mixture containing 12.5 μL 2 × SYBR Premix Ex Taq (TaKaRa), 50 ng cDNA template, 0.5 μL of each primer (10.0 μM) and 10.5 μL of double distilled H_2_O (dd H_2_O). The optimized operational procedure was performed as follows: 30 s at 95 °C (1 cycle), 5 s at 95 °C and 40 s at 60 °C (40 cycles) and then 60 s at 95 °C, 30 s at 55 °C and 30 s at 95 °C (1 cycle for melting curve analysis). Relative gene expression was evaluated as previously described [39].

### Generation of transgenic *Arabidopsis*

The pSUPER1300-35S-*GsoDA1* plasmids were constructed and were respectively transformed into *Agrobacterium tumefaciens* strain GV3101. Transformation of *Arabidopsis thaliana* ecotype Columbia (Col-0) was performed with a floral dipping method [40]. The transgenic plants were selected on Murashige & Skoog (MS) medium containing 40 mg/L hygromycin (Sigma-aldrich, USA), and confirmed further by PCR analysis.

### Analyses of transgenic plants

Seeds of Col-0 and T_3_ transgenic plants were sterilized by soaking in 70 % ethanol (v/v) for 5 min in 15 % NaClO (v/v) for 10 min. They were then rinsed four to five times with sterile ddH_2_O. The seeds were plated on Agar medium and incubated for 3 days at 4 °C in darkness. Half seeds of each line were plated onto solidified 1/2 MS medium containing 3 % (w/v) sucrose (pH = 5.8), and then were transferred to a growth chamber under long-day conditions (16 h light / 8 h dark at 23–25 °C). Another set of half seeds were plated on 1/2 MS medium containing various concentrations of NaCl (125 mM and 175 mM). After five days, germination rates were calculated. To evaluate the stress-tolerance of transgenic *Arabidopsis* seedlings, seeds were germinated and grown on 1/2 MS medium for one week, and the seedlings were transferred to soil to grow for three weeks before treatments. A total of 250 mM NaCl was imposed for 20 days (once for five days) until a lethal effect was observed on most of the wild-type plants. The content of chlorophyll at the 15th-day treatment and the aboveground dry biomass at the 30th-day treatment was measured.

### Determination of chlorophyll content

Rosette leaves of the Col-0 and the transgenic *A. thaliana* plants were collected and crushed in absolute alcohol. The mixture was rapidly shaken and left standing in the dark for 2 h. The debris was pelleted down by centrifugation and the supernatant was used for spectrophotometric determination. The optical densities of the supernatant were determined using a NanoDrop spectrophotometer (NanoDrop 2000, Thermo Fisher, USA). Chlorophyll contents were calculated using MacKinney’s specific absorption coefficients [41].

### Statistical analyses

Each experiment / measurement was performed using three independent biological replicates or repeated three times unless stated otherwise. A Student’s two-tailed *t*-test was used for statistical analysis in the present study.

### Sequencing analyses

The *DA1*-like genes in Williams 82 was characterized in our previous work [22]. The gene-specific primers were designed to get the full cDNA sequences in the soybeans Suinong 14 (*GmaDA1*) and ZYD00006 (*GsoDA1*). The unrooted phylogenetic tree was constructed using amino acid sequences. Both of the neighbor-joining (NJ) and maximum likelihood (ML; JTT model, bootstrap 100) methods were implemented in the software MEGA5 [42]. The tree was displayed using Treeview v0.4 [43]. In-frame insertions and deletions were detected by PROVEAN [44]. The consequence of sequence divergence between Gma-GsoDA1 orthologous pairs was predicted by SNAP and PROVEAN [44, 45]. All inserted fragments in the derived constructs were commercially sequenced in Beijing Genomic Institute (BGI, Beijing, China). Gene-specific primers for each analysis in the present work (Additional file 1: Table S3) were commercially synthesized in BGI (Beijing, China).

### Availability of supporting data

All relevant supporting data can be found within the additional files accompanying this article. Phylogenetic data supporting the results of this article are available in the TreeBASE repository at http://purl.org/phylo/treebase/phylows/study/TB2:S17527. Sequence data described in this article can be found in GenBank (http://www.ncbi.nlm.nih.gov) under the accessions of KR261655-KR261665 and KR349314-KR349324.

## Additional file

Additional file 1: Table S1. Sequence identities of GsoDA1 and GmaDA1 proteins. **Table S2.** Divergence of GsoDA1 and GmaDA1 proteins. Table S3. Primers used in the present work. **Figure S1.** Expression of the *GmaDA1* and *GsoDA1* genes in response to drought. **Figure S2.** Expression of the *GmaDA1* and *GsoDA1* genes in response to acid and base stresses. **Figure S3.** Expression of the *GmaDA1* and *GsoDA1* genes in response to ABA. **Figure S4.** PCR-confirmation of *35S::GsoDA1* transgenic *Arabidopsis.*1:** Figure S5.** Seed size of *35S::GsoDA1* transgenic *Arabidopsis*. **Dataset S1.** Soybean *DA1-*like expression in response to various abiotic stresses.

## Abbreviations

cDNA: Complementary DNA
qRT-PCR: Quantitative RT-PCR
ABA: Abscisic acid
SOS: Salt-overly-sensitive
NJ: Neighbor-joining
ML: Maximum likelihood
*GmaDA1*: *Glycine max DA1*
*GsoDA1*: *Glycine soja DA1*
