# Supplementary material for: Sequence and expression variations suggest an adaptive role for the DA1-like gene family in the evolution of soybeans
Source: BMC Plant Biol. 2015 May 15;15:120. doi: 10.1186/s12870-015-0519-0 (PMC4432951; doi:10.1186/s12870-015-0519-0)
Supplement: Additional file 1: Table S1. — Sequence identities of GsoDA1 and GmaDA1 proteins. Table S2. Divergence of GsoDA1 and GmaDA1 proteins. Table S3. Primers used in the present work. Figure S1. Expression of the GmaDA1 and GsoDA1 genes in response to drought. Figure S2. Expression of the GmaDA1 and GsoDA1 genes in response to acid and base stresses. Figure S3. Expression of the GmaDA1 and GsoDA1 genes in response to ABA. Figure S4. PCR-confirmation of 35S::GsoDA1 transgenic Arabidopsis.1: Figure S5. Seed size of 35S::GsoDA1 transgenic Arabidopsis. Dataset S1. Soybean DA1-like expression in response to various abiotic stresses. [file 12870_2015_519_MOESM1_ESM.pdf]

Table S1. Sequence identities of GsoDA1 and GmaDA1 proteins.

Gene	GmaDA 1-1 (561aa)	GmaDA 1-2 (532aa)	GmaDA 1-3 (485aa)	GmaDA 1-4 (547aa)	GmaDA 1-5 (473aa)	GmaDA 1-6 (526aa)	GmaDA 1-7 (471aa)	GmaDA 1-8 (481aa)	GmaDA 1-9 (530aa)	GmaDA 1-10 (481aa)	GmaDA 1-11 (473aa)
GsoDA1-1	**100**	77.3	47.7	92.8	46.4	75.4	47.4	57	77.4	57.4	47
GsoDA1-2	77.5	**98.3**	52.9	72.8	49.3	95.9	50.9	56.3	96.8	56.2	49.8
GsoDA1-3	47.8	52.1	**99**	46.1	73.5	50.7	94	49.7	52	48.7	75.1
GsoDA1-4	96.5	75.5	47.9	**95.8**	44.1	71.1	45.6	54.3	72.9	54.4	44.5
GsoDA1-5	46.4	49.2	73.6	45.7	**99.8**	48	75.1	50.2	49.1	49.4	91.6
GsoDA1-6	76.4	97.7	51.4	74.5	48.4	**98.5**	49.9	56.1	93.5	55.9	48.5
GsoDA1-7	47.4	51.1	94.1	47.2	74.6	50.4	**100**	50.7	50.4	49.7	76.4
GsoDA1-8	56.8	56.8	49.5	56.1	50	56.2	50.5	**99.8**	57.3	96.7	50
GsoDA1-9	77.4	96	52.2	75.5	49.1	94.7	50.4	57.2	**100**	56.9	49.6
GsoDA1-10	57.9	57.3	48.9	57	49.6	56.8	49.9	97.1	57.5	**99.4**	49
GsoDA1-11	46.9	49.7	75.2	46.2	91.6	49	76.9	49.8	49.6	49.2	**100**

Table S2. Divergence of GsoDA1 and GmaDA1 proteins.

GsoDA1/ GmaDA1 Proteins	Substitution (PROVEAN Score, prediction) #1	Substitution (SNAP prediction, expected accuracy) #2	Subcellular location	Indels (PROVEAN Score, prediction)
DA1-1			Nucleus	
DA1-2	D27G (-1.72, Neutral), E139G (0.82, Neutral), S159A (1.92, Neutral)	D27G (Neutral, 69%), E139G (Neutral, 89%), S159A (Neutral, 92%)	Vacuolar and nucleus	(460-464) SSSS/ (460-460) ---- (1.91, Neutral)
DA1-3	S12P (-1.53, Neutral), C18G (3.57, Neutral), I29R (0.29, Neutral), R31W (3.75, Neutral)	S12P (Neutral, 92%), C18G (Neutral, 69%), I29R (Neutral, 69%), R31W (Neutral, 92%)	Nucleus and cytomembrane	(427-427)- / (427-428)S (-0.68, Neutral)
DA1-4	E107Q (-0.56, Neutral), I115T (0.09, Neutral), C142R (0.35, Neutral), S250A (1.45, Neutral)	E107Q (Neutral, 69%), I115T (Neutral, 89%), C142R (Neutral, 90%), S250A (Neutral, 69%)	Nucleus	(118-139) EDEHLAKIQQEEEERL AKIQQE/ (118-118)---------------------(7.29, Neutral)
DA1-5	S88R (-1.58, Neutral)	R88S (Neutral, 69%)	Cytomembrane	
DA1-6			Vacuolar and nucleus	(38-45) VITLSSSN/ (38-38)-------- (3.64, Neutral)
DA1-7			Nucleus, endoplasmic reticulum and cytomembrane	
DA1-8	G299S (-5.7, deleterious)	G299S (Neutral, 60%)	nucleus	
DA1-9			Vacuolar and nucleus	
DA1-10	R67K (-0.18, Neutral), V160F (6.23, Neutral), V188I (0.95, Neutral)	R67K (Neutral, 96%), V160F (Neutral, 92%), V188 I (Neutral, 94%)	nucleus	
DA1-11			cytomembrane	

#1：PROVEAN, Protein Variation Effect Analyzer (http://provean.jcvi.org.). The cutoff value of PROVEAN was -2.5, when the score more than -2.5, the prediction was neutral; when the score was less than -2.5, the prediction was deleterious.
#2：SNAP, Screening for Non-Acceptable Polymorphisms (http:// www.rostlab.org/services/SNAP).

Table S3. Primers used in the present work.

Gene	Forward (5'-3')	Reverse (5'-3')	Usage
DA1-1	GACAGAATTTGGGATAATCAT	GCTGCGGTAATGGTCAATGTA	
DA1-2	TATGTGGTTGACTATGCGTA	TGGTGGGTGTAAAGGAAAGA	
DA1-3	CTCAGAAGGAGAAAGAGGATT	CCATACTTAGAGAACTTGCGT	
DA1-4	ATGAGGAGGATGAACATCTTG	ACTTTCATTGCTTCAGTTCCT	
DA1-5	CTATCATTTTCATTTTTCGTG	ACTCCAAACTCCTATGACAAC	
DA1-6	ATGGGTTGGTTTACTAAGTTG	TTTGAAAATAGATGATAT	cDNA isolation
DA1-7	ATGAAATGGCTTAATAAGCTT	ATTAGACTTGGACTTCCTCGT	
DA1-8	TTGAGTGAGTGAATGGGTTGG	GCAGCGGTTGGAAGTTGGTAA	
DA1-9	GTGTGGTTGACTATGTGTGT	GGTCCCTTACTTTGCTCTAT	
DA1-10	AAAGAGGATGCGGGTTATTAC	GATAGCAGAGGTTGGGAGTTG	
DA1-11	ATGAACCTTGTTTTTCATTGCT	CAAACTCCTATGAGAACGTGTC	
DA1-1	TGAGAAAGAAGACATTGACCG	AAGTTGCTCGTCTTCCTCAAG	
DA1-2	ACTTTAGAGAAATCAAATCCA	GCACATTTCATCACTCCTCAT	
DA1-3	TGGCTTGAGTCAGAAGTAAT	TCCATACTTAGAGAACTTGC	
DA1-4	TTCTCTGACCTATACAATGGC	ACTTTCATTGCTTCAGTTCCT	
DA1-5	GCTTCTTCCTCCTCTTACTC	CAGTCCAACCTTGTCTCTCA	
DA1-6	TGATGCTTTTGGTTGAGAGA	CTGGTATCCTGCTCCTATCCT	qRT-PCR
DA1-7	GGCTTGAGTCAGAAGTAATGC	AAACAAAGCACTTCTATCCAT	
DA1-8/10	ATACATTCACTCATCGCCTTC	GAATCAGCAGAGAGACAAGTT	
DA1-9	AAAGAGGATGCGGGTTATTAC	CGTTTCCATATCTGGGAGGAG	
DA1-11	ATCATGCTATCAGAGACTATT	TAACCTTTGAGGCGTAACCAA	
ACTIN	ATCTTGACTGAGCGTGGTTATTCC	GCTGGTCCTGGCTGTCTCC	
GsoDA1-1	ATACTGCAGAAATGGGTTGGTTTACCAAGT	ATAGGTACCATATGGAAAACTTCCTGTCAT	
GsoDA1-2	ATAAAGCTTTTTGCATGGGTTGGTTTACTAA	ATAACTAGTATATGGAAAACTTCCTGTCAAT	
GsoDA1-3	ATAACTAGTATGCAATGAAATGGCTCAGTA	ATAGGTACCTCCATACTTAGAGAACTTGCG	
GsoDA1-7	ATACTGCAGTCATGAAATGGCTTAATAAGC	ATAGGTACCCACTGGGAAAAAACCAGTCCA	Vector construct
GsoDA1-8	ATACTGCAGGAATGGGTTGGCTTAGCAGAAT	ATAACTAGTATATGGAAAAGTCCCTGTCATC	
GsoDA1-9	ATACTGCAGATTGCATGGGTGGGTTTACTA	ATAGGTACCATATGGAAAACTTCCGGTCAA	
GsoDA1-11	AAGCTTATAGTTTTATGAAATGGTTTGGTA	GGTACCATACAGTGGGAAATGACCAGTCAA	
SOS2	ATTGAGGCTGTAGCGAAC	GGTATTCCTTCTGTTGCC	
SOS3	GGAGGAATCTCTTCGCTG	CACGAAAGCCTTATCCACC	
FRY1	CGCAGTAGCACTAGGATTG	TTGACACCGAGTTTATTGG	qRT-PCR
P5SC1	TTCTCAGATGGTTTCCAGGTTG	TGGGAATGTCCTGATGGGTG	
ADH1	CTCTTGGTGCTGTTGGTTTAGG	AATTGGCTTGTCATGGTCTTTC	

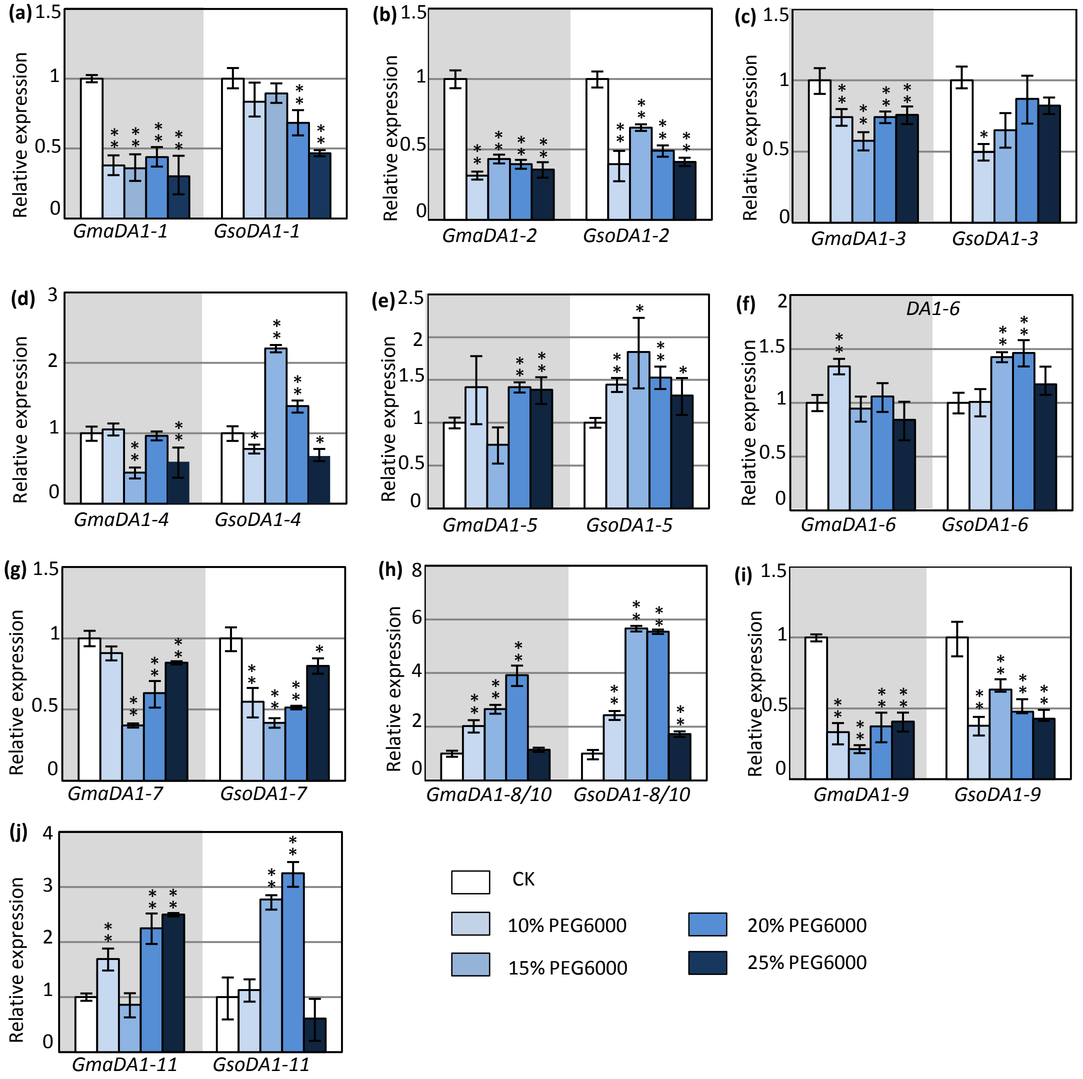

Figure S1. Expression of the *GmaDA1* and *GsoDA1* genes in response to drought.
Total RNAs from roots after 4 hrs treatment with different PEG6000 stresses were subjected to qRT-PCR analyses. Expressions of each gene in the non-treated conditions (white column) were set as controls (CK), while their expression variation in response to stresses is shown as indicated. The expression of *GmaDA1* genes in CK and 20% PEG6000 were shown previously [22]. The *ACTIN* gene was used as an internal control. The experiments were performed using three independent biological samples. Error bar: standard deviation. The * means significance at a $P < 0.05$ level, and the ** represent the significance at a $P < 0.01$ level.

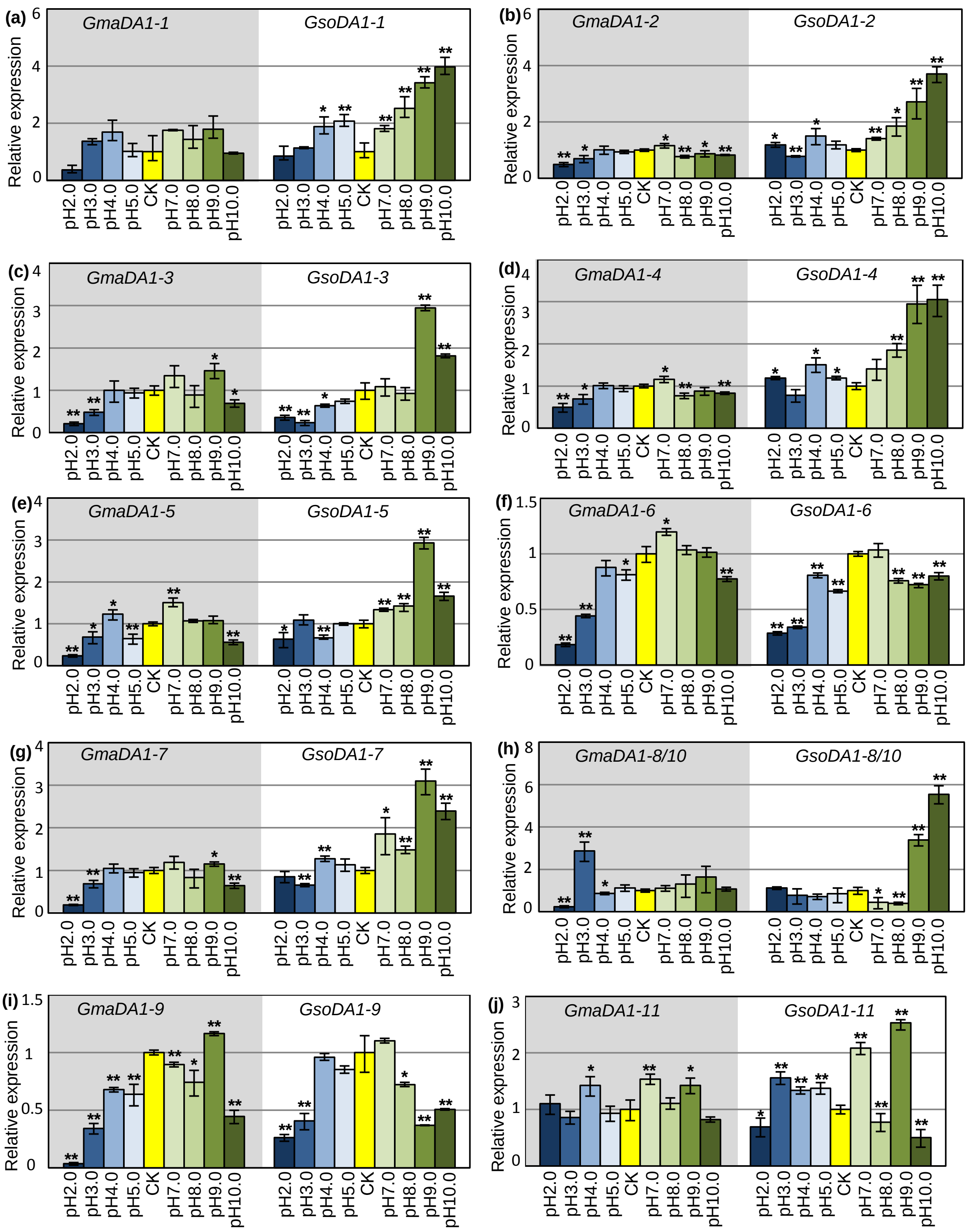

Figure S2. Expression of the *GmaDA1* and *GsoDA1* genes in response to acid and base stresses. Total RNAs from roots after 4 hrs treatment for different pH stresses were subjected to qRT-PCR analyses. Expressions of each gene in the non-treated conditions (yellow column) were set as controls (CK), while their expression variation in response to stresses are shown as indicated. The expressions of *GmaDA1* genes in CK, pH2.0 and pH10.0 were shown previously [22]. The *ACTIN* gene was used as an internal control. The experiments were performed using three independent biological samples. Error bar: standard deviation. The * means significance at a $P < 0.05$ level, and the ** represents the significance at a $P < 0.01$ level.

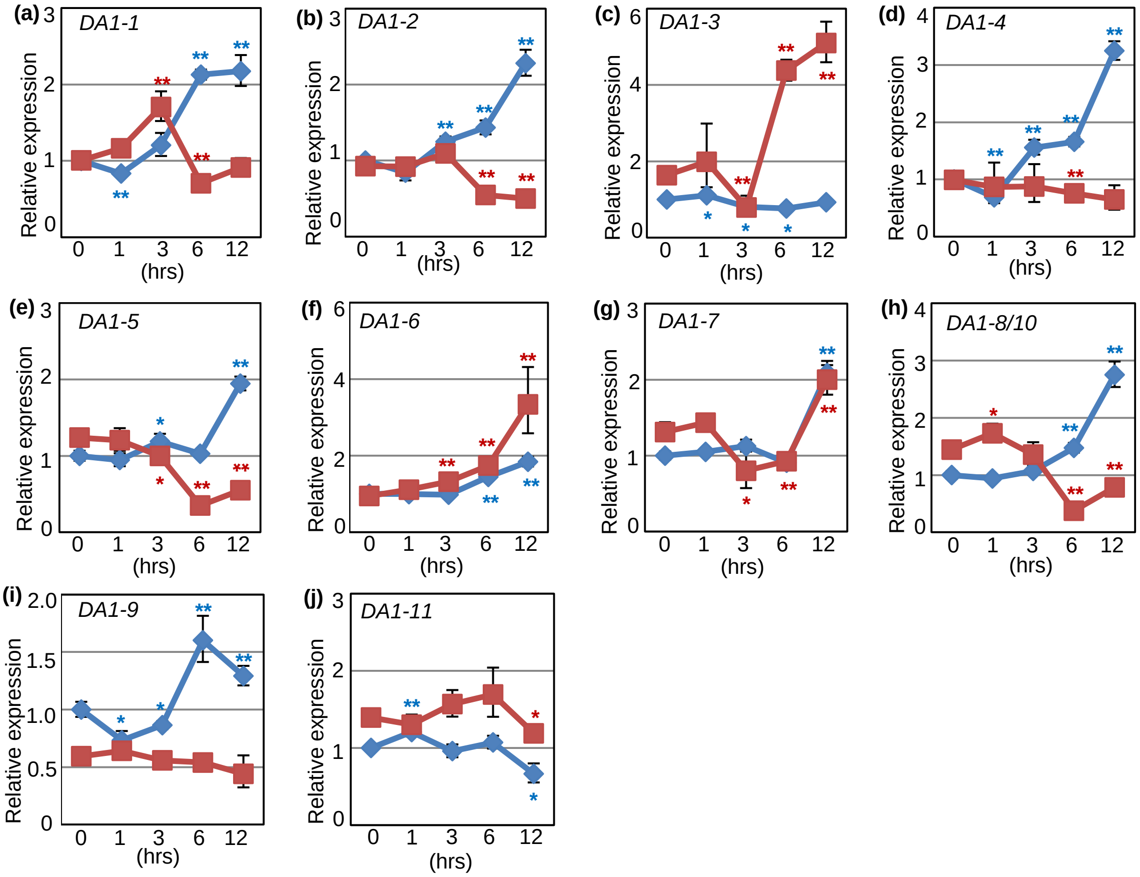

Figure S3. Expression of the *GmaDA1* and *GsoDA1* genes in response to ABA.
Total RNAs from roots at 0 hr, 1 hr, 3 hrs, 6 hrs and 12 hrs after ABA treatment were subjected to qRT-PCR analyses. Expression of each gene in the non-treated (0 hr) was set as a control (CK) and the *ACTIN* gene was used as an internal control. The expressions of *GmaDA1* genes (in blue) [22] and *GsoDA1* genes (in red) were compared. The experiments were performed using three independent biological samples. Error bar: standard deviation. The * means significance at a $P < 0.05$ level, and the ** represent the significance at a $P < 0.01$ level.

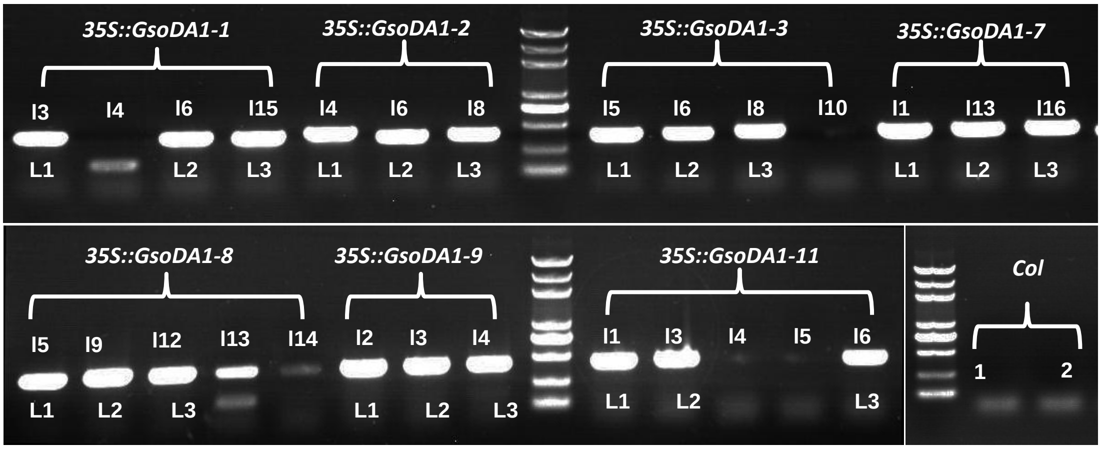

Figure S4. PCR-confirmation of *35S::GsoDA1* transgenic *Arabidopsis.*

The genomic DNA of transgenic and wild-type *Arabidopsis* plants was used. The specific primer pair 1 (cttccaatcactgattat; gcattcatgagtatccat) was used for the detection of *35S::GsoDA1-1*, *35S::GsoDA1-2*, *35S::GsoDA1-8* and *35S::GsoDA1-9*, and the specific primer pair 2 (ttgtcagggaagcatcca; agcagattccatgcactc) was used for the detection of *35S::GsoDA1-3*, *35S::GsoDA1-7* and *35S::GsoDA1-11* transgenic *Arabidopsis* lines. No bands were amplified from wild-type *Arabidopsis*. The l4 in *GsoDA1-1,* l10 in *GsoDA1-3,* l13 and l14 in *GsoDA1-8,* and l4 and l5 in *GsoDA1-11* were the false positives and were not further analyzed. Three true transgenic lines for each construct were renamed as L1, L2 and L3, respectively.

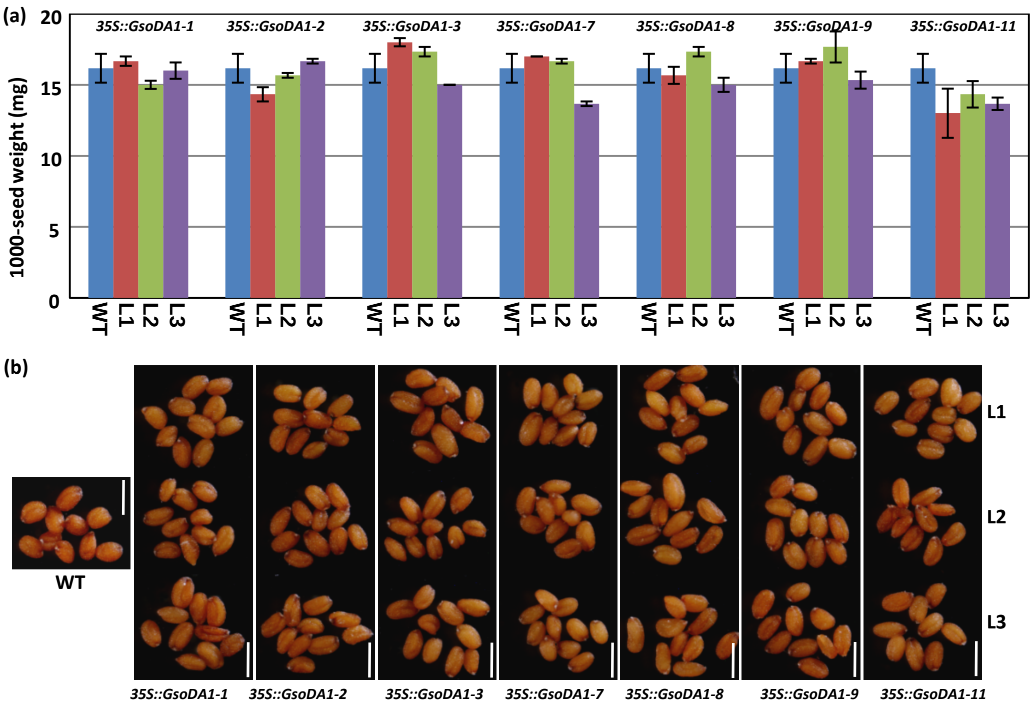

Figure S5. Seed size of *35S::GsoDA1* transgenic *Arabidopsis*.
(**a**) Average seed weights of the wild type (WT, blue), and transgenic *Arabidopsis* (red, green and purple for L1, L2 and L3) are shown as milligrams (mg) per 1,000 seeds. Bars are standard deviations. (**b**) Seeds of WT and *35S::GsoDA1* transgenic lines. Bars = 0.5 mm.

Dataset S1. Soybean *DA1*-like expression in response to various abiotic stresses.
In order to maximally exploit the adaptive role of the soybean *DA1* genes, we also investigated gene expression in response to other external abiotic stresses, such as drought, acidic/alkali-stresses and abscisic acid (ABA) stresses. To create a stress condition, the wild and cultivated soybean seedlings were treated with different chemicals, and then qRT-PCR was used to evaluate gene expression (See Methods).

Drought
A large set of qRT-PCR analyses revealed that the *GmaDA1* and *GsoDA1* orthologous pairs basically had a similar changing tendency for gene expression in response to the drought stress mimicked by different concentrations of PEG6000 (Fig. S1). These *DA1*-like genes were either up-regulated or down-regulated; however, different *Gma-GsoDA1* orthologous pairs had different and fine expression trends. The mRNA transcripts of *DA1-1*, *DA1-2*, *DA1-3*, *DA1-7* and *DA1-9* were down-regulated significantly between the wild and cultivated soybeans (Fig. S1a–c, g, i). The expression levels of *DA1-2* and *DA1-9* showed a sustained decrease ($P < 0.002$) (Fig. S1a, i), while the expression levels of *DA1-1*, *DA1-3* and *DA1-7* remained repressed only in some concentrations of PEG6000 in the wild and cultivated soybeans (Fig. S1b, c, g). In contrast, the expressions of *DA1-5*, *DA1-6*, *DA1-8/10* and *DA1-11* were strongly induced by some PEG6000 treatments (Fig. S1e, f, h, j). Only the expression of the *Gma-GsoDA1-4* orthologous pair had a different responding tendency. Besides the downregulation related to 10% and 25% PEG6000, the transcript amount of *GsoDA1-4* was also observed to be up-regulated by 15% and 20% PEG6000, while the transcript level of *GmaDA1-4* was either down-regulated or not changed (Fig. S1d). Similarly, when the soybean *DA1* paralogs in a species, like either *GmaDA1* or *GsoDA1*, were compared, they had different responding patterns (Fig. S1).

Acidic and alkali-stresses
The results of qRT-PCR analyses of the soybean *DA1-like* genes in response to acidic and alkali-stresses were compared (Fig. S2). The transcripts of *Gma-GsoDA1* orthologous pairs had a conserved accumulated pattern under the acid and alkali stresses except for *DA1-1* ($P = 0.02$); however, the expression of the *DA1* paralogs in soybeans were strikingly diverged ($P < 1.93E\text{-}4$). Under the alkali stresses, the expression levels of all the wild soybean *DA1-1*, *DA1-2*, *DA1-3*, *DA1-4*, *DA1-5* and *DA1-7* genes were to some extent significantly up-regulated with a different magnitude ($P < 0.002$), but in the cultivated soybean, except that the *GmaDA1-1* was basically unchanged, the other *DA1*-like genes were all firstly induced and then were repressed (Fig. S2a–e, g). *Gma-GsoDA1-6* and *Gma-GsoDA1-9* were basically repressed under the alkali stresses (Fig. S2f, i). The expression of *GmaDA1-8/10* was essentially unchanged ($P > 0.2$), while the *GsoDA1-8/10* was repressed at pH7.0 and pH8.0 ($P < 0.03$), and then significantly induced at pH9.0 and pH10.0 ($P < 0.0002$) (Fig. S2h). The expression levels of *Gma-GsoDA1-11* shared a similar but various pattern in response to different pH values (Fig. S2j). The expression patterns were also varied in the acidic stresses (Fig. S2). *DA1-3*, *DA1-6* and *DA1-9* had consistent expression patterns in wild and cultivated soybeans. All of them were repressed to some extent ($P < 0.04$; Fig. S2c, f, i). However, the other *Gma-GsoDA1* orthologous gene pairs had various expression patterns in response to acidic stresses (Fig. S2a, b, d, e, j).

ABA
We also examined the effect of ABA on soybean *DA1*-like expression (Fig. S3). The expression of *DA1* genes diverged in wild and cultivated soybeans, especially after treatment for 3 hrs (Fig. S3). These genes included *DA1-1*, *DA1-2*, *DA1-3*, *DA1-4*, *DA1-5*, *DA1-8/10* and *DA1-9*. The divergences consisted of two categories. The *DA1-3* was up-regulated in the wild soybean and down-regulated in the cultivated soybean (Fig. S3c), while the variation of the *DA1-1*, *DA1-2*, *DA1-4*, *DA1-5*, *DA1-8/10* and *DA1-9* genes were opposite (Fig. S3a, b, d, e, h, i). The remaining *DA1-6*, *DA1-7* and *DA1-11* genes had a consistent expression tendency in wild and cultivated soybeans, in which both of the *DA1-6* and *DA1-7* genes were up-regulated, but the *DA1-11* gene was down-regulated (Fig. S3f, g, j). Nonetheless, the soybean *DA1* paralogs in a species were statistically mostly conserved ($P > 0.29$).
